# Association of *SLC2A9* genotype with phenotypic variability of serum urate in pre-menopausal women

**DOI:** 10.3389/fgene.2015.00313

**Published:** 2015-10-14

**Authors:** Ruth K. Topless, Tanya J. Flynn, Murray Cadzow, Lisa K. Stamp, Nicola Dalbeth, Michael A. Black, Tony R. Merriman

**Affiliations:** ^1^Department of Biochemistry, University of OtagoDunedin, New Zealand; ^2^Department of Medicine, University of OtagoChristchurch, New Zealand; ^3^Department of Medicine, University of AucklandAuckland, New Zealand

**Keywords:** genotype, exposure, interaction, urate, *SLC2A9*, variance, gout, uric acid

## Abstract

The *SLC2A9* gene, that encodes a renal uric acid reuptake transporter, has genetic variants that explain ∼3% of variance in urate levels. There are previous reports of non-additive interaction between *SLC2A9* genotype and environmental factors which influence urate control. Therefore, our aim was to further investigate the general phenomenon that such non-additive interactions contribute to genotype-specific association with variance at *SLC2A9*. Data from 14135 European individuals were used in this analysis. The measure of variance was derived from a ranked inverse normal transformation of residuals obtained by regressing known urate-influencing factors (sex, age, and body mass index) against urate. Variant *rs6449173* showed the most significant effect on serum urate variance at *SLC2A9* (*P* = 7.9 × 10^-14^), which was maintained after accounting for the effect on average serum urate levels (*P* = 0.022). Noting the stronger effect in a sub-cohort that consisted of pre-menopausal women and younger men, the participants were stratified into males and pre-menopausal and post-menopausal women. This revealed a strong effect on variance in pre-menopausal women (*P* = 3.7 × 10^-5^) with a weak effect in post-menopausal women (*P* = 0.032) and no effect in men (*P* = 0.22). The T-allele of *rs6449173*, which associates with increased urate levels, was associated with the greater variance in urate. There was a non-additive interaction between *rs6449173* genotype and female gender in control of serum urate levels that was driven by a greater increase in urate levels associated with the T-allele in women. Female hormones, and/or other factors they influence or are associated with (such as iron levels, temperature, testosterone) interact with *SLC2A9* genotype in women to determine urate levels. The association of *SLC2A9* with greater variance in pre-menopausal women may reflect the cyclical changes resulting from menstruation.

## Introduction

Heterogeneity in genetic variance exists when the effect a genotype has on phenotype is influenced by external factors. Such factors include differing environmental exposures, internal factors (such as epistatic interactions with other genetic variants), or other biological phenomena. An example of the latter are the stochastic processes underlying photoreceptor choice of cone cells in developing tri-chromatic vision or increased variation with aging within individuals of a given genotype (Jacobs, 2009; Paré et al., 2010; Geiler-Samerotte et al., 2013). In humans, genotypic control of phenotypic variability has been demonstrated for *FTO/IRX3* in body mass index (BMI; Yang et al., 2012), *LEPR* in C-reactive protein levels and *ICAM1* and *PNPLA3* in soluble ICAM1 levels (Paré et al., 2010). A total of 23 genome-wide significant variance expression quantitative trait loci single nucleotide polymorphisms (SNPs) have been reported in lymphoblastoid cell lines, of which ∼70% could be attributed to non-additive gene by environment (GxE) interactions (Brown et al., 2014).

Urate is a medically important metabolite. Elevated serum urate (hyperuricemia) is a central cause of gout, the most common form of inflammatory arthritis characterized by severe pain, disability, and joint damage. A genome-wide association study (GWAS) has demonstrated that levels of serum urate are influenced by genetic variants in 28 loci, with the strongest effects observed in renal and gut transporters of uric acid (Köttgen et al., 2013). In particular, variants in *SLC2A9* have a very large effect on urate levels (e.g., *rs12498742*) and gout [e.g., *rs11942223*; in strong linkage disequilibrium (LD) with *rs12498742*], explaining 2–3% of the variance in serum urate in European individuals and a substantially stronger effect in women than in men (Hollis-Moffatt et al., 2009; Köttgen et al., 2013). Sex is the strongest reported interacting variable with *SLC2A9* genotype to control urate levels (*P* = 8.2 × 10^-6^ for sex, *P =* 0.02–0.03 for age and alcohol intake, *P* > 0.38 for BMI, diabetes and hypertension status; Voruganti et al., 2014).

In addition, non-additive interactions between *SLC2A9* genetic variants, food items, and diuretic medication have been reported. The influence of diet and diuretic medication on serum urate is well-established. Use of diuretics and consumption of seafood, red meat, alcohol and sugar-sweetened beverage (SSB) and tomatoes all associate with increased urate and the risk of gout (Choi and Curhan, 2004, 2008; Choi et al., 2004a,b, 2005, 2008, 2012; Rasheed et al., 2013; Batt et al., 2014; Flynn et al., 2015). Non-additive interaction between *rs6449173* (in strong LD with *rs12498742* and *rs11942223*) genotype at *SLC2A9* and SSB consumption in control of serum urate and risk of gout has been reported (Batt et al., 2014). A similar interaction between *rs6449213* genotype (in strong LD with *rs6449173*) and alcohol has been reported in American Indian individuals (Voruganti et al., 2014). There is evidence that *SLC2A9* (*rs13129697*) and *SLC22A11* (*rs2078267*) genotype interact with diuretics to determine the risk of gout (McAdams-DeMarco et al., 2013), although this was not replicated in a larger study (Bao et al., 2015). Whilst these findings require further validation, the data suggest that non-additive gene–environment interactions are involved in control of urate levels at *SLC2A9*. Such interactions are important to understand in order to increase insight into the molecular pathogenesis of hyperuricemia.

To further investigate non-additive interactions between *SLC2A9* genotype and environmental exposures in control of urate levels and risk of gout a series of classical interaction tests focused on putative instrinsic and extrinsic interactors could be conducted as has been performed previously (McAdams-DeMarco et al., 2013; Batt et al., 2014; Voruganti et al., 2014). Alternatively, because an interacting genotype would be expected to result in larger variance (Paré et al., 2010; Struchalin et al., 2010), a single dimensional analysis for genotypes influencing phenotypic variance could be used. Therefore, the aim of this study was to test for association with variance in serum urate at *SLC2A9* and potentially identify other environmental interactions with *SLC2A9* in serum urate.

## Materials and Methods

### Participants

Participants of European ancestry were included from five separate sample sets (**Table 1**). Two were from the Atherosclerosis Risk in Communities study (ARIC; *n* = 5362) and the Framingham Heart Study (FHS Generation 3; *n* = 3282) from which people taking antihypertensive or urate-lowering medication, or who self-reported physician-diagnosed kidney disease or gout were excluded. Two were from the Coronary Artery Risk Development in Young Adults study (CARDIA; *n* = 1496) and the Cardiovascular Health Study (CHS; *n* = 2799), from which individuals taking urate-lowering medication and who self-reported physician-diagnosed kidney disease or gout were excluded. ARIC individuals self-reporting as taking diuretics (*n* = 1196) were also included as the fifth sample set. No individuals were excluded based on estimated glomerular filtration rate (eGFR) – there were 47 (0.33%) individuals with eGFR < 30, 46 of whom were from CHS and one from ARIC. The research procedures were in accordance with the ethical standards of the institutional review boards relevant to the various data sets. Written informed consent was given by all participants. The ARIC, FHS, CHS, and CARDIA analyses (project #834) were approved by the relevant Database of Genotype and Phenotype^1^ Data Access Committees. The overall project was approved by the New Zealand Health and Disability Ethics Committee (ref: 05/10/130).

**Table 1:** Demographic and clinical details of the three data sets, and associations of clinical features with serum urate concentrations.

	FHS		ARIC		ARIC diuretics		CARDIA		CHS	
		*r^2^*^∧^		*r^2^*		*r^2^*		*r^2^*		*r^2^*
Serum urate (*SD*), mmol/L	0.308 (0.086)	–	0.332 (0.080)	–	0.398 (0.097)	–	0.284 (0.087)	–	0.328 (0.087)	–
Age (*SD*), years	39.4 (8.64)	8.51 × 10^-5^	53.5 (5.58)	0.0062	55.9 (5.55)	0.015	40.7 (3.33)	4.55 × 10^-5^	72.4 (5.47)	4.75 × 10^-4^
Females, % (n)	53.8 (1765)	0.44	54.2 (2903)	0.29	65.7 (786)	0.15	53.7 (803)	0.39	61.1 (1710)	0.11
BMI (*SD*), kg/m^2^	26.4 (5.18)	0.16	26.0 (4.33)	0.14	29.2 (5.71)	0.070	27.1 (5.85)	0.14	26.2 (4.42)	0.088
Post-menopausal women, % (*n*)^∗^	9.0 (159)	0.0066	49.8 (1447)	0.022	51.0 (401)	0.017	5.2 (42)	0.0050	89.4 (1528)	–
PC1	–	3.04 × 10^-5^	–	1.82 × 10^-5^	–	3.75 × 10^-5^	–	7.69 × 10^-7^	–	6.84 × 10^-5^
PC2	–	1.48 × 10^-4^	–	4.56 × 10^-5^	–	1.44 × 10^-4^	–	5.93 × 10^-6^	–	2.25 × 10^-4^

*^∗^Excluding 90 FHS, 942 ARIC, 266 ARIC diuretic, 234 CARDIA, and 182 CHS participants for which menopausal status was unknown, intermediate or were receiving hormone replacement therapy. The *r^2^* value is the proportion of variance in urate explained by menopause in women with known menopause status.*

*^∧^*r^2^* values, representing the proportion of variance in serum urate explained by the variable, were obtained by linear regression of urate against the variable listed, using the lm function in R, and extracting the Multiple *R^2^* value from the output summary.*

**SD*, standard deviation; PC, principal component.*

### Phenotypes

Phenotypes from baseline exams were used for all studies with the exception of CARDIA, where phenotypes from exam six were used. For the total 7967 European female participants menopause status was determined by self-report. Those who were pregnant, breastfeeding, taking hormone replacement therapy, or did not report menopause status were excluded from the menopause analysis. Subjects who reported as post-menopausal, but had menstruated in the last 12 months were also excluded from the menopause analysis. Serum urate levels were measured using a standard uricase assay (precision value of 8.6%) in the ARIC and CARDIA datasets (Henry et al., 1957; ARIC Investigators, 1989; Dyer et al., 1999). CHS used a Kodak Ektachem 700 analyzer with reagents (Eastman Kodak, Rochester, NY, USA), which had a coefficient of variation of 2.4% (Cushman et al., 1995). A phosphotungstic acid reagent autoanalyzer was used to measure serum urate levels in the FHS data set participants (Crowley, 1964). This method has a precision value of 2.8% (Henry et al., 1957; Crowley, 1964).

### Genotypes

Publicly available genome-wide genotype data (Affymetrix 6.0) from the ARIC and CARDIA data sets, combined Affymetrix 50K and 500K platform data from the FHS data set and CHS genotypes imputed from Illumina Human CNV370v1 was used to impute the full *SLC2A9* region (±200 kb) using Impute2 version 2.3.0 with the 1000 Genomes Phase 1 integrated variant set phased with SHAPEIT2 as the reference haplotype panel (Delaneau et al., 2014).

### Statistical Analysis

Analysis was done using the R statistical software package (version 3.2^2^). R code is presented in Supplementary Table S1.

The variable used as a measure of variance was derived from residuals obtained from sex- and cohort-specific analysis regressing age and BMI (BMI causally affects urate levels Lyngdoh et al., 2012; Palmer et al., 2013). The top two principal component eigenvectors (calculated using default parameters with SMARTPCA Patterson et al., 2006) were also included to account for cryptic relatedness within sample sets. A ranked inverse normal transformation of the absolute residual values yielded the *z*-score, with the *z^2^*-score being the variance variable. The inverse normal transformation, while likely to be overly conservative, minimizes a possible mean-variance relationship of phenotype (Yang et al., 2012). To account for the influence of the mean effect of *rs6449173* genotype on the variance effect, using the approach of Yang et al. (2012), the genotype-specific mean urate was subtracted from the urate level of each individual participant and the genotype effect on variance was retested on squared residuals as described above. Data sets were combined by inverse-variance weighted meta-analysis in R (meta version 4.2-0^3^) using a fixed effects model, except where there was evidence for heterogeneity (*P*_Het_ < 0.05) whereupon a random effects model was used.

Interaction analysis between menopausal status and *rs6449173* was conducted using the R lm function with a linear model regressing urate against age, BMI, *rs6449173* allele, menopausal status and the interaction term between menopause and *rs6449173* allele. Post-menopausal and pre-menopausal women were compared to men (as the referent group) in separate models and the effect of the interaction term reported.

## Results

**FIGURE 1:**
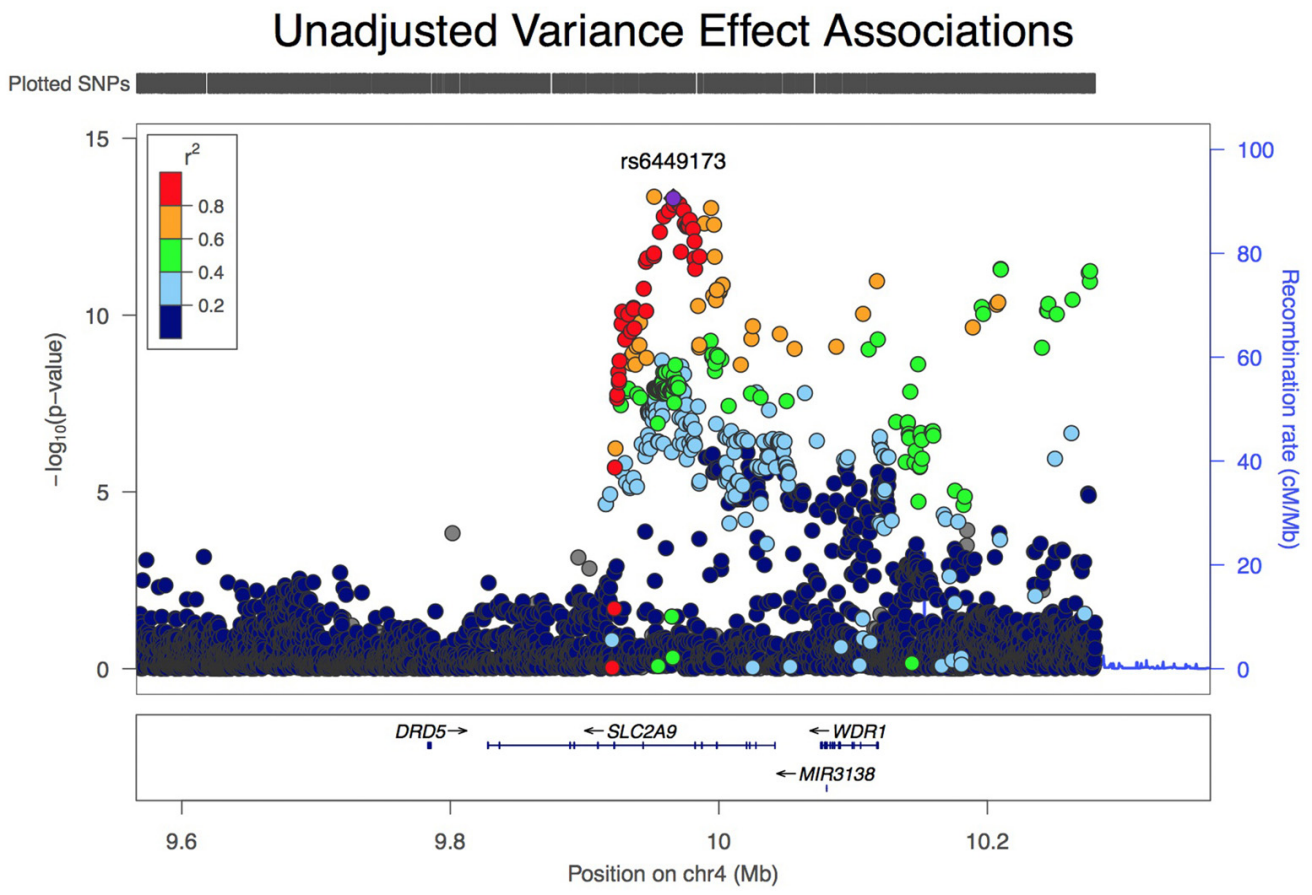
**LocusZoom view of association of 5544 variants at the *SLC2A9* locus with variance, unadjusted for effect on mean urate level**.

Analysis of all *SLC2A9* variants within ±200 kb of the gene for association with variance in serum urate levels resulted in a single association peak (**Figure 1**). We chose to analyze SNP *rs6449173* which is one of a large number (*n* = 136) of SNPs (**Supplementary Figure S1**) in a haplotype block including the variant (*rs12498742, r*^2^ = 0.96 with *rs6449173*) previously reported as most strongly associated with average serum urate by GWAS (Köttgen et al., 2013). *Rs6449173* demonstrated the strongest effect on serum urate variance at this locus (**Figure 1**; **Table 2**; β_Tallele_ = -0.152, *P* = 7.9 × 10^-14^). The initial region-wide analysis (**Figure 1**) was unadjusted for the possible confounding effect of genotype-specific mean urate levels. After adjustment of the mean effect the genotype-specific effect on the variance was reduced in magnitude, and the direction of effect was reversed, with the major T allele associated with greater variance in urate (β_Tallele_ = 0.047, *P* = 0.022).

**Table 2:** The influence of accounting for the *rs6449173* average effect on the estimated serum urate variance effect.

	*N*	β^∗^	*SE*	*P*
**ARIC**	5356			
Mean effect		0.023	0.002	8.7 × 10^-56^
Unadjusted variance effect		-0.153	0.033	4.1 × 10^-6^
Adjusted variance effect		0.024	0.033	0.47
**FHS**	3282			
Mean effect		0.023	0.002	2.7 × 10^-38^
Unadjusted variance effect		-0.192	0.042	5.8 × 10^-6^
Adjusted variance effect		0.063	0.043	0.14
**ARIC diuretics**	1196			
Mean effect		0.031	0.004	2.5 × 10^-12^
Unadjusted variance effect		-0.117	0.073	0.11
Adjusted variance effect		0.043	0.073	0.56
**CHS**	2799			
Mean effect		0.023	0.003	2.0 × 10^-19^
Unadjusted variance effect		-0.156	0.045	4.7 × 10^-4^
Adjusted variance effect		-0.016	0.045	0.72
**CARDIA**	1496			
Mean effect		0.026	0.003	2.6 × 10^-22^
Unadjusted variance effect		-0.079	0.062	0.20
Adjusted variance effect		0.208	0.061	7.3 × 10^-4^
**Combined**	14129			
Mean effect		0.024	0.00095	4.7 × 10^-140^
Unadjusted variance effect		-0.152	0.020	7.9 × 10^-14^
Adjusted variance effect		0.047	0.020	0.022

*^∗^Mean effect units mmol/L urate; variance effect is estimate of allelic additive effect on *z^2^*.*

**FIGURE 2:**
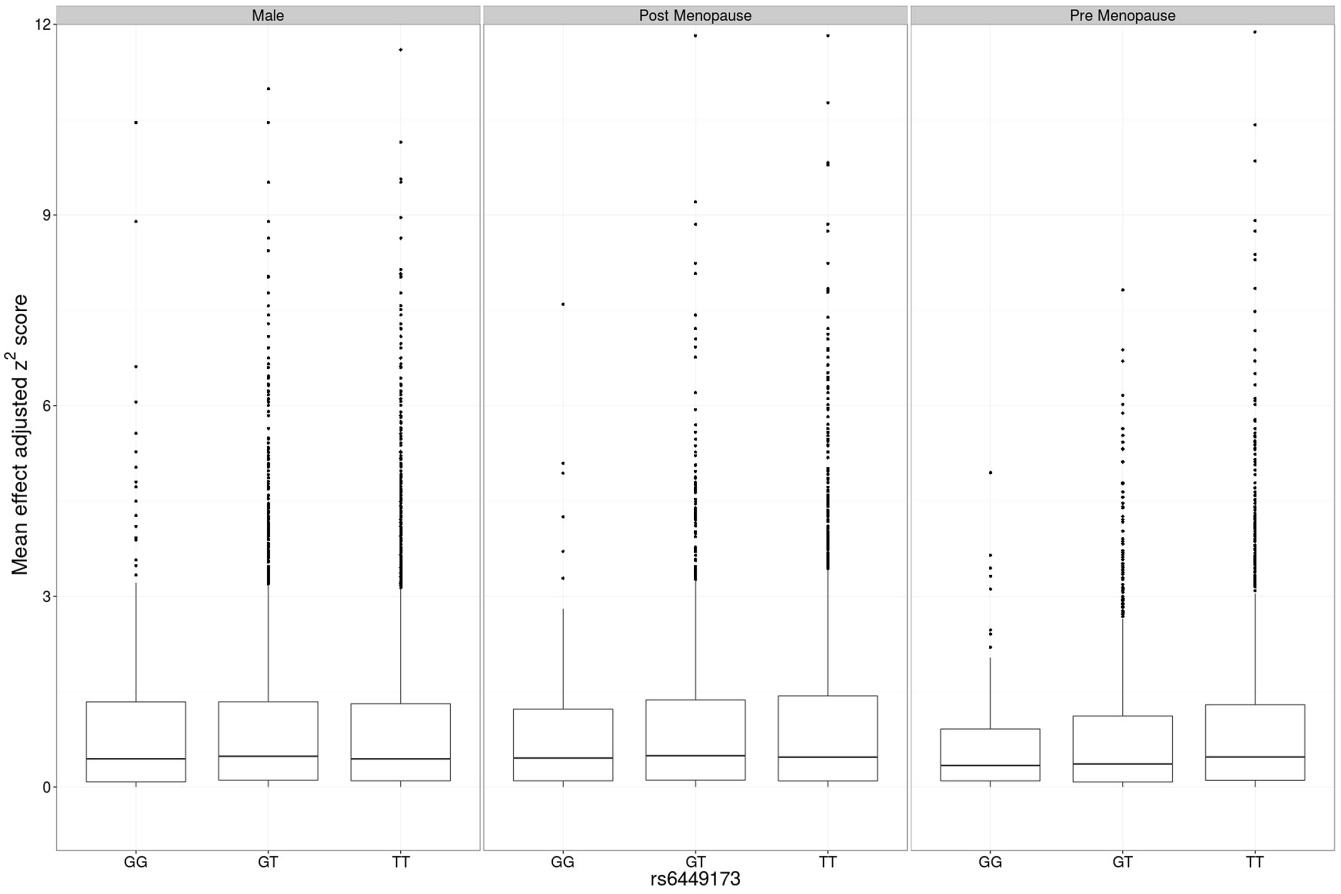
**Main effect adjusted *z*^2^-scores at *rs6449173*.** The genotype-specific median (standard deviation) *z*^2^ for men was GG 0.439 (1.458), GT 0.487 (1.443), TT 0.441 (1.380), for post-menopausal women was GG 0.411 (1.103), GT 0.465 (1.304), TT 0.451 (1.470), and for pre-menopausal women was GG 0.408 (0.891), GT 0.373 (1.215), TT 0.507 (1.514).

The adjusted variance effect was statistically significant only in the CARDIA data set (**Table 2**; β = 0.208, *P* = 7.3 × 10^-4^; all other European sample sets β ≤ 0.043, *P* ≥ 0.14). Noting that this sample set was comprised entirely of younger individuals (**Table 1**; men and predominantly pre-menopausal woman); noting that the effect of *SLC2A9* on average urate levels is stronger in women (Köttgen et al., 2013); and noting the association of menopause with serum urate levels (Hak and Choi, 2008) we therefore reanalyzed the *SLC2A9* genotype effect on variance in men and pre-menopausal and post-menopausal women separately. This revealed that the variance effect was stronger in pre-menopausal women in the combined sample set (**Table 3**; β = 0.191, *P* = 3.7 × 10^-5^) than post-menopausal women (β = 0.087, *P* = 0.032) or men (β = -0.038, *P* = 0.22). The variance effect was visualized using box plots (**Figure 2**). This showed that increased median *z*^2^-scores and increased standard deviation were observed with the TT-genotype and decreased median *z*^2^-scores and standard deviation were associated with the GG genotype in pre-menopausal women. This effect was less obvious in post-menopausal women and was not observed in men.

**Table 3:** Association of *rs6449173* genotype with serum urate variance in sample sets stratified into men, pre-menopausal women and post-menopausal women, with adjustment for *rs6449173* mean effect.

		*N*	Variance β^∗^	*SE*	*P*	*r^2^* #
ARIC	Total	5356	0.024	0.033	0.47	0.00010
	Men	2453	-0.053	0.050	0.28	0.00047
	Pre-menopausal women	515	0.188	0.105	0.074	0.00624
	Post-menopausal women	1447	0.083	0.063	0.19	0.00119
FHS	Total	3282	0.063	0.043	0.14	0.00067
	Men	1517	-0.008	0.063	0.89	0.00001
	Pre-menopausal women	1513	0.165	0.062	7.6 × 10^-3^	0.00470
	Post-menopausal women	159	-0.015	0.187	0.93	0.00004
ARIC diuretics	Total	1196	0.043	0.073	0.56	0.00029
	Men	410	-0.088	0.126	0.49	0.00119
	Pre-menopausal women	90	0.430	0.271	0.12	0.02811
	Post-menopausal women	401	0.102	0.128	0.43	0.00159
CHS	Total	2799	-0.016	0.045	0.72	0.00005
	Men	1089	-0.121	0.073	0.097	0.00253
	Pre-menopausal women	0	–	–	–	–
	Post-menopausal women	1528	0.093	0.062	0.13	0.00150
CARDIA	Total	1496	0.208	0.061	7.3 × 10^-4^	0.00762
	Men	693	0.112	0.092	0.23	0.00211
	Pre-menopausal women	527	0.231	0.100	0.022	0.00997
	Post-menopausal women	42	0.269	0.369	0.47	0.01304
Combined^∧^	Total	14129	0.047	0.020	0.022	0.00037
	Men	6162	-0.038	0.031	0.22	0.00024
	Pre-menopausal women	2645	0.191	0.046	3.7 × 10^-5^	0.00637
	Post-menopausal women	3577	0.087	0.040	0.032	0.00129

*^∗^Variance effect is estimate of allelic additive effect on z^2^.*

*^∧^Combined by meta-analysis using a fixed effects model.*

*# *r^2^* values, representing the proportion of variance in variance in serum urate explained by *rs6449173*, were obtained by linear regression of the adjusted *z*^2^ score against *rs6449173* using the lm function in R, and extracting the Multiple *R^2^* value from the output summary.*

The hypothesis that there was non-additive interaction between genotype and female hormone status in determining serum urate (adjusting for age and BMI) was evaluated. Men and post-menopausal women have similar estrogen levels and estrogen has a similar paracrine role, not acting solely as an endocrine factor produced by the ovaries in each group (Khosla et al., 1998; Simpson and Davis, 2001). We therefore expected pre-menopausal women to have an interaction effect of greater magnitude than post-menopausal women (when both groups are compared to men), reflective of the variance results. However, allelic interaction terms were of approximately equal effect for both pre-menopausal and post-menopausal women as compared to men (β_Interaction_ = 0.013 mmol/L, *P* = 2.4 × 10^-6^ and β_Interaction_ = 0.012 mmol/L, *P* = 3.5 × 10^-6^, respectively). This effect was driven by a greater increase in serum urate levels when the T allele is present in pre-menopausal women (β_TT_ – β_GG_ = 0.075) and post-menopausal women (β_TT_ – β_GG_ = 0.065) compared to men (β_TT_ – β_GG_ = 0.038; **Table 4**).

**Table 4:** Association by linear regression and interaction of *rs6449173* genotype with average serum urate levels in pre-menopausal women and post-menopausal women and men stratified by genotype.

*rs6449173* genotype	GG β (mmol/L), *P*	GT β (mmol/L), *P*	TT β (mmol/L), *P*	Interaction term β_interaction_ (mmol/L), *P*
Men	0.000 (Reference)	0.021, 1.9 × 10^-4^	0.038, 2.1 × 10^-15^	0.000 (Reference)
Post-menopausal women	-0.088, <2.0 × 10^-16^	-0.047, <2.0 × 10^-16^	-0.023, 6.1 × 10^-6^	0.012, 3.51 × 10^-6^
Pre-menopausal women	-0.135, <2.0 × 10^-16^	-0.090, <2.0 × 10^-16^	-0.060, <2.0 × 10-^16^	0.013, 2.37 × 10^-6^

*Adjusted by age and BMI.*

We included an interaction term in the variance model (adjusted for mean effect) resulting in β_Interaction_ = 0.228, *P* = 4.49 × 10^-5^ for pre-menopausal women and β_Interaction_ = 0.125, *P* = 0.015 for post-menopausal women, both with men as referent group. In this model, for pre-menopausal women the proportion of variance (*r*^2^) in phenotypic variance explained by adding the interaction term increased from 0.00018 to 0.0021 and in post-menopausal women increased from 0.000011 to 0.00062. The increase in *r^2^* indicates that non-additive interaction with menopausal status also contributes to the observed association between *rs6449173* and variance in urate levels. This interaction is stronger in pre-menopausal than post-menopausal women. This phenomenon is separate to the non-additive interaction between *rs6449173* and sex *per se* in determining mean urate levels (**Table 4**).

## Discussion

We present evidence that the *SLC2A9* genotype associated with average serum urate levels also differentially associates with variance in urate levels in pre-menopausal women. This may reflect the cyclical changes resulting from menstruation. There was also non-additive interaction between sex and *SLC2A9* in determining urate levels, replicating the findings of Voruganti et al. (2014). We interpret these findings to indicate that the intrinsic biological phenomenon of female hormones (which change upon menopause) and/or other factors that they directly affect (such as temperature, iron levels, testosterone) interact with *SLC2A9* genotype in a non-additive fashion in women to determine urate levels. The effect of the *rs6449173* T-allele in raising urate is greater in women.

Our data can be compared to the findings of Yang et al. (2012) who associated *FTO/IRX3* with genotype-specific variance in BMI. This locus, like *SLC2A9*, has the strongest mean effect size on phenotype in the genome. At the *FTO* SNP *rs7202116* the allelic effect on average phenotype did not contribute to the observed effect on variance, in contrast with *SLC2A9 rs6449173* where the allelic effect on mean phenotype contributed considerably to the genotype-specific association with variance in phenotype. Stratifying the sample set in our study clarified the analysis and clearly showed a genotype-specific effect on variance in urate in pre-menopausal women after accounting for the average effect. While a number of changes occur throughout the menstrual cycle (e.g., iron levels, temperature, estrogen and progesterone levels) the factors with the most evidence supporting a role in urate control are iron levels (Ghio et al., 2005; Mainous et al., 2011) and hormones. Female hormones (estrogens) increase the fractional excretion of uric acid and reduce serum urate levels (Yahyaoui et al., 2008). Our data are consistent with a model whereby female hormones contribute directly via *SLC2A9* in a genotype-specific fashion both to the mean urate levels and variance in urate levels. In animals estrogen reduces renal urate reabsorption by reducing *Slc2a9* protein levels (Takiue et al., 2011), so it is conceivable that in human females estrogen could contribute to the *SLC2A9*-mediated mean effect by a *rs6449173* genotype-specific effect on expression of *SLC2A9*.

In pre-menopausal women urate levels vary across the menstrual cycle with endogenous estradiol associated with reduced, and follicle stimulating hormone associated with increased, urate (Mumford et al., 2013). Thus, also in a genotype specific manner, female hormones would be expected to contribute to variance potentially owing to the cyclical changes in levels of female hormones in pre-menopausal women or other factors influenced or associated with menstrual cycling in pre-menopausal women (e.g., oral contraceptive use Stöckl et al., 2012). Whilst estrogen levels in post-menopausal women are more similar to levels in men than pre-menopausal women (Khosla et al., 1998), and serum urate levels rise to levels approximately equivalent to those of men after menopause, the data in **Table 4** suggest that post-menopausal women and men still control serum urate levels differently. However we were unable to test for a direct interaction between female hormone levels and *SLC2A9* genotype. Owing to the use of cross-sectional data we were also unable to test for any genotype-specific effect on intra-individual variability in pre-menopausal women. Such a study would allow some evaluation of the hypothesis that *SLC2A9* genotype interacts non-additively with female hormones or another variable factor associated with menstruation.

The association with variance was largely restricted to pre-menopausal women. There is epidemiological evidence from cross-sectional observational data that menopause associates (independent of measured confounders) with increased urate, that post-menopausal hormone replacement therapy associates with reduced urate (Simon et al., 2006; Hak and Choi, 2008; Stöckl et al., 2012) and that estrogen levels are inversely associated with urate levels during the menstrual cycle (Mumford et al., 2013). This is consistent with clinical studies demonstrating a urate-lowering effect of hormone replacement therapy (Nicholls et al., 1973; Gotfredsen et al., 1983; Sumino et al., 1999), however, there is little definitive evidence that this effect occurs through an influence on renal uric acid handling (Nicholls et al., 1973; Gotfredsen et al., 1983; Antón et al., 1986; Ghio et al., 2005). The increased urate-associated TT genotype of *rs6449173* drives the association with variance in urate in European pre-menopausal women (**Figure 2**), suggesting that understanding the molecular consequence of the genetic effect that this allele tags is key to understanding the mechanism for the observed genotype-specific effects of *SLC2A9* on average urate and variability in urate. To this end, determining if *rs6449173* is in fact associated with the separate *SLC2A9* isoforms (full length and missing 28 cytoplasmic residues), as published data suggest (Döring et al., 2008; Vitart et al., 2008), will be important.

There are multiple independent effects at *SLC2A9* with the urate association signal at *SLC2A9* encompassing 100s of extremely strongly associated genetic variants over a very large region (500 kb; Köttgen et al., 2013). In a GWAS of serum urate levels in East Asians (Okada et al., 2012), the strongest genome-wide association with urate was at *SLC2A9*, but with a different SNP variant (*rs3775948*). The most strongly associated European variant [*rs12498742*, in strong LD (*r^2^* = 0.86) with *rs6449173*; Köttgen et al., 2013] was not associated in the East Asian GWAS probably because of the rarity of the minor allele (prevalence of ∼1%). Interestingly the rs3775948 mean effect in East Asians also has, by conditional analysis, an effect in Europeans independent of the European mean effect (Stahl et al., 2014). Furthermore, a GWAS testing for association of common copy number variation with serum urate in Europeans (Scharpf et al., 2014) found association with two copy number variations 200 and 350 kb upstream of *SLC2A9* that were each genetically independent of the *rs12498742* effect at *SLC2A9*. Thus there is evidence for at least three independent variants in *SLC2A9* that influence urate levels in Europeans, and a separate variant in East Asians. The study of Wei et al. (2014) is consistent with the above studies in providing evidence for multiple independent genetic effects at the *SLC2A9* locus – five independent genetic effects were reported. Additional complexity in genetic control of urate levels at *SLC2A9* was revealed with epistasis between genetic variants at the *SLC2A9* locus influencing urate levels. [Note that *rs6449173* and SNPs in strong LD were not amongst SNP pairs in Wei et al. (2014) exhibiting epistasis.] Combined with the evidence here for a genotype-dependent effect at *SLC2A9* on variance, previous reports of non-additive GxE interaction at *SLC2A9* (McAdams-DeMarco et al., 2013; Batt et al., 2014; Voruganti et al., 2014) and evidence for a population-specific influence of genotype to fructose response (Dalbeth et al., 2013), it is clear that this is an extremely complex urate and gout locus that will be very challenging to understand using genetic epidemiology.

The contribution of non-additive GxE interactions to the phenomenon of ‘missing’ heritability (predicted genetic variance not explained by genome-wide studies assessing the contribution of common genetic variants) is unclear, although it has been suggested that a failure to include the possibility of interactions in an inheritance model can lead to over-estimation of the genetic heritability of a phenotype (Manolio et al., 2009; Zuk et al., 2012). Urate levels are an ideal phenotype to address this question given that there are established dietary and drug environmental exposures (see Introduction) that have relatively immediate temporal effects on urate levels via hepatic production and perhaps also by interfering with excretion (Dalbeth and Merriman, 2013; Batt et al., 2014). This means that data on environmental exposures that are likely causal of changes in urate levels are able to be collected at the same time as phenotype in cross-sectional study designs. To facilitate identification of non-additive GxE interactions, systematically identifying genetic variants with a genotype-specific effect on variance in phenotype, in genome-wide approaches using very large sample sets and accounting for the average effect, can prioritize variants that can be tested for non-additive GxE with specific environmental exposures in linear and logistic models that incorporate interaction terms. Furthermore, identification of variance-associated genetic variants could allow identification of new urate loci which may have average main effects obscured in genome-wide studies that do not incorporate environmental exposures.

## Conflict of Interest Statement

The authors declare that the research was conducted in the absence of any commercial or financial relationships that could be construed as a potential conflict of interest.

## Footnotes

1 http://www.ncbi.nlm.nih.gov/gap

2 http://www.R-project.org/

3 http://CRAN.R-project.org/package=meta

## Supplementary Material

Figure S1 **Haploview plot depicting intermarker linkage disequilibrium of 136 *SLC2A9* variants associated with mean serum urate level by Köttgen et al. (2013) that include *rs6449173* (right arrow) and the variant with the strongest association, rs12498742 (left arrow).** Linkage disequilibrium range in the haplotype block is from 0 to 1, mean is 0.55, *SD* is 0.36.
