# Supplementary material for: Association of SLC2A9 genotype with phenotypic variability of serum urate in pre-menopausal women
Source: Front Genet. 2015 Oct 14;6:313. doi: 10.3389/fgene.2015.00313 (PMC4604317; doi:10.3389/fgene.2015.00313)
Supplement: Supplementary file 1 [file Table_1.DOCX]

**Supplemental Table S1**

*R code*

Mean (main) effect urate association analysis

URATE = SNP + Predictors + residuals

Unadjusted urate variance analysis

URATE = Predictors + residuals

then(Inverse normal transformed residuals)^2 correlated to SNP genotype

Main effect adjusted urate variance analysis

Adjusted urate = Predictors + residuals.

then (Inverse normal transformed residuals)^2 correlated to SNP genotype

Where Adjusted urate = individual urate measurements with the mean urate subtracted for each genotype group

Script in R for all 3 analyses

MALES<-subset(DATASET, SEX==1)

FEMALES<-subset(DATASET, SEX==2)

#Create mean urate by SNP and adjusted urate by SNP columns

library(dplyr)

MALES<-MALES %>% group_by(RS6449173) %>% mutate(meanuratebysnp= mean(URATE), adjuratebysnp=(URATE-meanuratebysnp))

FEMALES<-FEMALES %>% group_by(RS6449173) %>% mutate(meanuratebysnp= mean(URATE), adjuratebysnp=(URATE-meanuratebysnp))

#Store of residuals and conversion to Z2 scores for both adjusted and unadjusted urate tests

MALESunadj<-lm(URATE~AGE+BMI+PCA1+PCA2, data=MALES)

summary(MALESunadj)

MALES$unadjres=MALESunadj$residuals

MALES$unadjZ <- qnorm((rank(MALES$unadjres,na.last="keep")-0.5)/sum(!is.na(MALES$unadjres)))

MALES$unadjZ2 <- (MALES$unadjZ)^2

MALESadj<-lm(adjuratebysnp~AGE+BMI+PCA1+PCA2, data=MALES)

summary(MALESadj)

MALES$adjres=MALESadj$residuals

MALES$adjZ <- qnorm((rank(MALES$adjres,na.last="keep")-0.5)/sum(!is.na(MALES$adjres)))

MALES$adjZ2 <- (MALES$adjZ)^2

FEMALESunadj<-lm(URATE~AGE+BMI+PCA1+PCA2, data=FEMALES)

summary(FEMALESunadj)

FEMALES$unadjres=FEMALESunadj$residuals

FEMALES$unadjZ <- qnorm((rank(FEMALES$unadjres,na.last="keep")-0.5)/sum(!is.na(FEMALES$unadjres)))

FEMALES$unadjZ2 <- (FEMALES$unadjZ)^2

FEMALESadj<-lm(adjuratebysnp~AGE+BMI+PCA1+PCA2, data=FEMALES)

summary(FEMALESadj)

FEMALES$adjres=FEMALESadj$residuals

FEMALES$adjZ <- qnorm((rank(FEMALES$adjres,na.last="keep")-0.5)/sum(!is.na(FEMALES$adjres)))

FEMALES$adjZ2 <- (FEMALES$adjZ)^2

COHORT<-rbind(MALES, FEMALES)

# Urate to RS6449173 association

testCOHORT<-lm(URATE~RS6449173+SEX+AGE+BMI+PCA1+PCA2, data=COHORT)

summary(testCOHORT)

# Unadjusted urate variance association

COHORTunadj<-lm(unadjZ2~RS6449173, data=COHORT)

summary(COHORTunadj)

# Adjusted for mean urate variance association

COHORTadj<-lm(adjZ2~RS6449173, data=COHORT)

summary(COHORTadj)

Script in R for non-additive interaction analysis

lm(URICACID~as.numeric(RS6449173)*MENOPAUSE+AGE+BMI,subset=MENOPAUSE!="PRE",data=all_cohorts)

lm(URICACID~as.numeric(RS6449173)*MENOPAUSE+AGE+BMI,subset=MENOPAUSE!="POST",data=all_cohorts)
