# Supplementary figures and images for: Association of SLC2A9 genotype with phenotypic variability of serum urate in pre-menopausal women
Source: Front Genet. 2015 Oct 14;6:313. doi: 10.3389/fgene.2015.00313 (PMC4604317; doi:10.3389/fgene.2015.00313)

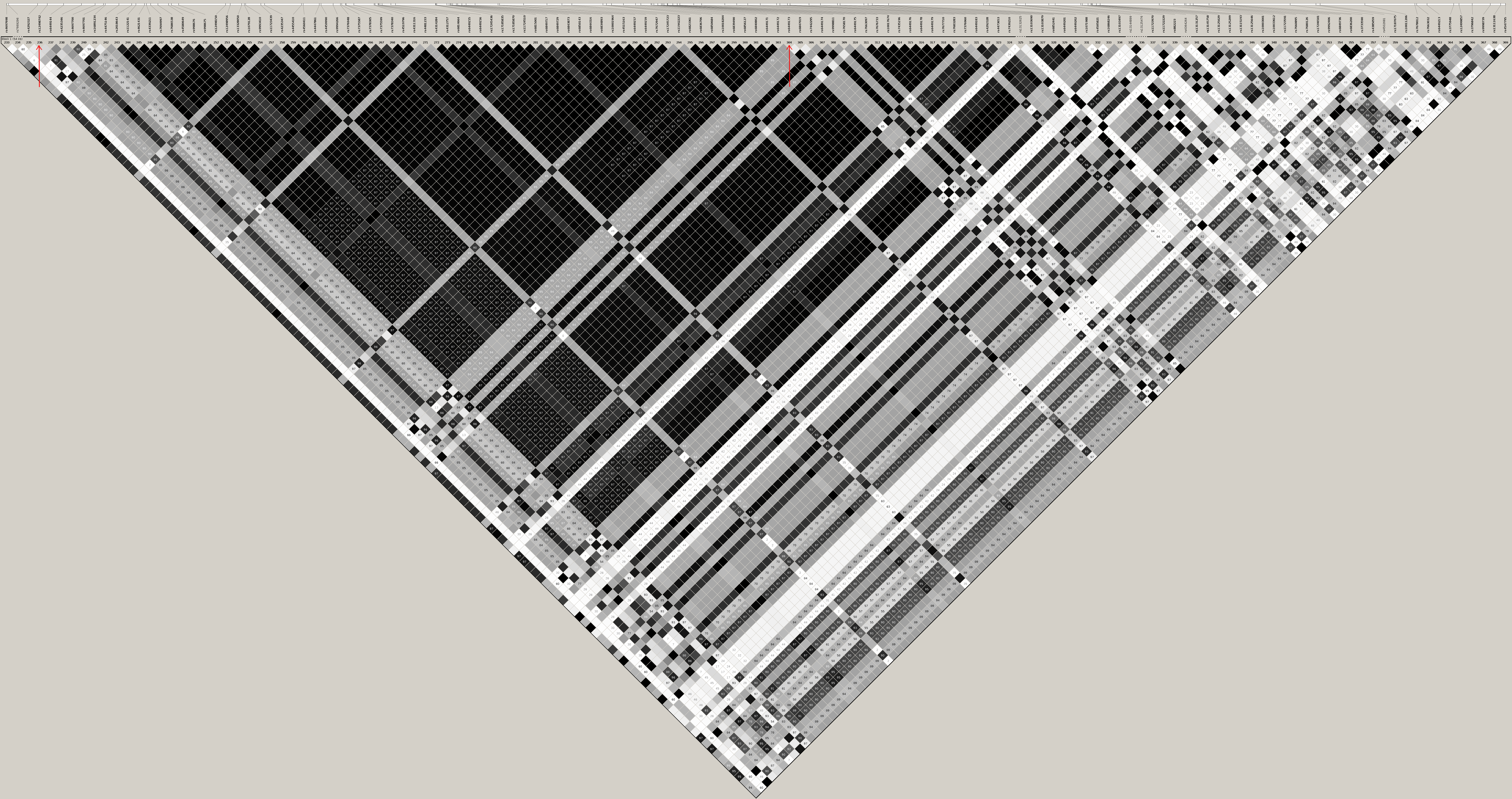

Supplement: Figure S1 — Haploview plot depicting intermarker linkage disequilibrium of 136 SLC2A9 variants associated with mean serum urate level by Köttgen et al. (2013) that include rs6449173 (right arrow) and the variant with the strongest association, rs12498742 (left arrow). Linkage disequilibrium range in the haplotype block is from 0 to 1, mean is 0.55, SD is 0.36. [file Image_1.PNG]
